# Aluminum as a Base for Artificial Dentures

**Published:** 1890-02

**Authors:** A. Hugh Hipple

**Affiliations:** Stratford.


ARTICLE III.

### ALUMINUM AS A BASE FOR ARTIFICIAL DENTURES.

BY A. HUGH HIPPLE, D. D. S., STRATFORD.

(Read before the Ontario Dental Society.)

The dental profession has been looking for many years and is still looking for a new base upon which to construct artificial dentures. Gold is so expensive as to be beyond the reach of many patients. It is, moreover, somewhat difficult to manipulate, and under even the most favorable circumstances owing to the necessity of swaging it, only an approximate adaptation to the mouth can be obtained. Silver, while cheap enough to be within the reach of all, has the other disadvantage of gold, and besides tarnishes so readily in the presence of sulphur or sulphuretted hydrogen that it should rarely if ever be used for that purpose. Vulcanized India rubber has come to be the almost universal cheap base, and being cheap and requiring only a small amount of skill in its manipulation to produce fair results, the great majority of dentists appear to be perfectly satisfied with it and do not care to look for anything better. The better class of practitioners, however, have been constantly on the lookout for some material to take its place, possessing its advantages without its disadvantages.

Richardson in his *Mechanical Dentistry* says:—"While there are undoubtedly many important uses to which vulcanized india rubber may be applied in the practical department of dentistry, and for which it would be difficult to find an adequate substitute, yet there are accumulating evidences leading to the conclusion that its total abandonment as a base for artificial dentures, by intelligent and conscientious practitioners everywhere, is an event of the not far distant future."

Of the different substitutes for vulcanite which have been proposed and employed, none to my mind presents so many advantages and so few disadvantages as aluminum, particularly since by the aid of certain simple appliances it can be cast directly upon a plaster model. As it is only within the last few years that the properties of this wonderful metal have been studied and understood, a general description of it, together with an outline sketch of some of its possibilities, may not be out of place.

To begin with, then, aluminum is a silver-white metal which has a fine lustre, and is capable of receiving a bright polish. Although not found free in nature it is yet the most abundant of all metals. It is an essential constituent of more than 200 different minerals, and good compact clay is found upon analysis to contain from 10 to 20 per cent of the pure metal. One of its most striking peculiarities is its lightness. Its specific gravity is 2.56, so that it is only one-third as heavy as iron, less than one-fourth as heavy as silver, and about one-eighth as heavy as gold. It is very malleable and very ductile. It posesses about the same hardness as silver but is more tenacious. It is one of the best conductors of heat and electricity known, being eight times a better conductor than iron, and almost equal to silver. It differs from silver, however, in resisting entirely the action of sulphur or sulphuretted hydrogen. It melts at a temperature slightly higher than the melting point of zinc and therefore within easy reach. It does not oxidize in air, and is unaffected by water at any temperature. It is unacted upon by vegetable acids, and even strong sulphuric acid is said not to attack it. In fact it is one of the most unalterable of the metals; but may be dissolved in hydrochloric acid. It is tasteless, odorless, and absolutely harmless, and no bad results can possibly follow in its use in the mouth.

The abundance of this metal and the necessity for cheap methods of extracting it from its compounds, become evident to us when we consider that a cubic yard of clay weighs about 4000 lbs., and contains from 400 to 900 lbs. of pure aluminum. As $12.00 per lb. may be considered as the present commercial value of the metal, the total value of the aluminum contained in a single cubic yard of clay amounts to at least $5.000. It seems almost incredible but it is a fact nevertheless, that society is willing to pay from $5,000 to $10,000 for the purified aluminum contained in a single cubic yard of ordinary clay! Is it any wonder then in view of the prize which awaits the inventor of a cheap process of reducing aluminum, that nearly every chemist in the land has devoted more or less attention to the matter, and that, as a recent writer puts it, "nearly eveything that is possible has been tried and nearly everything that is impossible has been proposed."

The method of extracting the metal which has been employed in the past is known as Deville's method, and depends upon the action of metallic sodium upon a double chloride of aluminum and sodium. As sodium is an expensive metal, and as it requires more than three pounds of sodium to make a pound of aluminum, this method is necessarily very expensive. Electric methods are also being tried with some success, but what promises to be the most important of all is that of Dr. Netto, of Dresden. The Krupp iron works at Essen, in Germany, are introducing his processes, and state confidently that they will be able to supply ingots of this metal at a cost not much greater than that at which steel bars were produced forty or fifty years ago. If this is true, in view of its abundance and valuable properties, it is not unreasonable to expect to see in the near future this shining white metal taking the place of iron and steel. It is as tenacious as malleable, and as ductile—when soft—as iron, and yet becomes almost as hard as steel when hammered and rolled. Then too, it will not corrode or disappear in rust like those metals. There seems to be no reason why it should not take the place of the iron work of passenger and freight cars, with a two-thirds reduction in weight, and a great saving in wear and tear of rails and track. The great ocean steamers if sheathed with this metal would not only be much lighter and have their tonnage proportionately increased, but their sides would be practically indestructible from the action of the elements. In the construction of bridges the fact of posessing the maximum of tensile strength and indestructibility, combined with lightness and flexibility, gives it a decided advantage over any other material, while for electrical purposes especially in the case of suspended telegragh wires, all heavy metals will have to give way as its conducting capacity is nearly equal to copper. The experiment of using an alloy of aluminum in the manufacture of cannon has been tried with very satisfactory results, although its cost has heretofore prevented its general adoption. Once it becomes cheap, however,—and it is only a question of time when it will become so—on account of its being only one-third the weight of iron, the ease with which large guns can be handled will give it a decided advantage over steel, bronze, or any like metal. It was this fact and the hope of being able to use it in naval warfare which influenced the Emperor Napoleon III. to extend his patronage to Deville, while the latter was carrying on his experiments. It is interesting to note, too, in connection with the patronage of this emperor, that the first article ever manufactured of aluminum was a baby's rattle, which was intended for the unfortunate Prince Imperial, who afterward lost his life in Zululand.

As the methods for its extraction become more perfect and its cheapness increased, may we not expect to see houses even built of hollow moulded bricks of aluminum, instead of bricks of clay, and these houses filled with furniture carved and moulded from this beautiful and indestructible metal? The world has had its stone age, its bronze age, and its steel age. Is it not possible that it may now be entering a new aluminum age, which will be as much in advance of the present as the bronze was in advance of the stone?

The lightness, conductivity, malleability, and other valuable properties of this remarkable metal would seem to make it a very desirable base upon which to construct artificial dentures, and it has beed used for that purpose with more or less success. Swaged aluminum plates have been constructed, but the difficulty of finding a suitable solder has never been entirely overcome. Probably the best solder for this purpose is Dr. Starr's, composed of seven parts aluminum to one of pure tin. Another alloy used for soldering aluminum is composed of 90 parts tin, 5 parts bismuth, and 5 of aluminum. Thus far, however, the attempts made to attach teeth to swaged aluminum plates by means of solder have been far from satisfactory. Vulcanite attachments have yielded better results, and very desirable dentures can be constructed in that way. It is found that rubber vulcanized in contact with pure aluminum becomes intimately adherent to it, and as the aluminum plate on account of its lightness can be made two or three times as thick as a gold plate, very strong dentures can be constructed in that way. Another and still more promising form of aluminum work is that known as "cast aluminum." One of the first and most successful experimenters in this direction was Dr. Bean, of Baltimore. who patented a very ingenious process for casting aluminum plates. The process, however, was difficult and somewhat unceitain in its results. The chief difficulties to be overcome in the casting of aluminum depend upon the lightness, and its lack of fluidity when melted. Dr. Bean attempted to overcome these by means of a tall column of melted metal, which, acting by its own weight, forced the metal in the flask into all the irregularities of the mould. Unfortunately while ascending Mt. Blanc in 1870, this experimenter was killed by an avalanche and his method never came into general use.

Dr. Carroll, of meadville, Pa., has invented a process for casting aluminum which in the hands of skillful mechanics has been eminently successful. The sluggishness of the metal when melted is overcome by pneumatic pressure, which forces it into the finest crevices of the mould. The outfit made use of by him consists of an automatic gas furnace, a specially constructed flask, and a pneumatic plumbago crucible, with accessory apparatus. In casting, the flask containing the mould is heated in the furnace along with the crucible containing the metal, and when the metal is melted, the crucible is placed in position over the flask, and the melted aluminum is forced into the mould by pneumatic pressure from a rubber bulb. By this means an almost exact reproduction of the wax base-plate can be obtained, and as the aluminum files easily, it can be trimmed to any shape and polished almost as readily as a rubber plate.

Just to what extent these cast aluminum plates are likely to take the place of vulcanite in the near future I am not prepared to say, but I believe that when the advantages of aluminum as a base and the simplicity of the process come to be recognized, the use of vulcanite as a base for artificial dentures will be abandoned by a large number of dentists. I am led to believe this because aluminum possesses not only the ordinary advantages of a metallic over a plastic base, but has several important advantages peculiar to itself. Among the most important are:—

1. Exceeding lightness—a full upper denture weighing usually from ¼ to ½ oz.

2. Perfect adaptation—being cast directly upon the model of the mouth.

3. An unusual degree of conductivity.

4. Perfect freedom from oxidation.

5. Compatibility with the tissues of the mouth.

6. Great stiffness combined with strength and durability.

7. Simplicity of the process of manufacture.—*Dominion Dental Journal.*

